# Social support and psychological distress of patients with pituitary adenomas: chain-mediated effects of self-efficacy and rumination

**DOI:** 10.3389/fpsyg.2025.1564736

**Published:** 2025-04-15

**Authors:** Lating Zhang, Na Cheng, Shan Zhang, Xinhui Liang, Yao Jia, Xue Jiang

**Affiliations:** ^1^School of Nursing, Shaanxi University of Chinese Medicine, Xianyang, Shaanxi, China; ^2^Department of Neurosurgery, Tangdu Hospital, Fourth Military Medical University, Xi'an, Shaanxi, China; ^3^Xijing 986 Hospital Department, Fourth Military Medical University, Xi'an, Shaanxi, China; ^4^School of Nursing, Air Force Medical University, Xi'an, Shaanxi, China; ^5^Department of Anesthesiology, Tangdu Hospital, Fourth Military Medical University, Xi'an, Shaanxi, China

**Keywords:** social support, psychological distress, pituitary adenomas, self-efficacy, rumination

## Abstract

**Introduction:**

Psychological distress in patients with pituitary adenomas affects their quality of life and is influenced by various aspects such as sociocultural factors, personal resources, and stressors. Therefore, the aim of this study was to investigate the chain-mediated role of self-efficacy and rumination in the relationship between social support and psychological distress.

**Methods:**

The study was based on the Wilson-Cleary model of health-related quality of life and self-efficacy theory. We investigated 500 patients with surgically treated pituitary adenomas in three tertiary hospitals in Shaanxi Province, China. We used the “Social Support” and “General Self-Efficacy” scales, “Rumination Scale,” and the “Psychological Distress in Patients with Pituitary Adenomas” questionnaire to conduct the survey, and the data were analyzed using structural equation modeling and bootstrap methods to examine the relationships between the variables.

**Results:**

The results showed that there was a significant positive correlation between social support and self-efficacy, social support and self-efficacy negatively predicted rumination and psychological distress, respectively. Rumination significant positive correlation with psychological distress, and social support, indirectly affected psychological distress through the chain-mediated effect of self-efficacy and rumination. This represents a compound multiple mediating effect on psychological distress.

**Discussion:**

Clinical practitioners should enhance social support for patients with pituitary adenomas, improve patients’ self-efficacy, and mitigate rumination to reduce patients’ psychological distress.

## 1 Introduction

Pituitary adenomas are common benign intracranial tumors originating from adenopituitary cells, constituting approximately 10–15% of intracranial tumors, and are the most common endocrine and nervous system tumors (Dai et al., 2021a; Daly and Beckers, 2020). Surgical removal is the mainstay of treatment for pituitary adenomas. The mass-occupying effect of pituitary adenomas and the resulting hormonal disruption can lead to symptoms involving multiple organ systems throughout the body (Dai et al., 2021b; Strickland et al., 2021). This type of disease is common in young adults; once the disease seriously affects the patient’s growth, development, and ability to work and is accompanied by significant clinical manifestations, that active treatment of the patient’s symptoms is does not entirely improve, and some patients will develop a chronic disease that requires long-term medication to control, which significantly compromises both physical and mental health (Luo, 2020; Sheng, 2020).

Psychological distress is an emotional experience of extreme inner discomfort caused by various factors, which includes psychological (cognitive, behavioral, and emotional), social, spiritual, or physical experiences, and can lead to psychological disorders ranging from common vulnerability, sadness, fear, anxiety, depression, pain, and social isolation (Riba et al., 2019). To draw clinical attention to psychological distress, the International Psycho-Oncology Association identified it in 2010 as the sixth vital sign after temperature, respiration, pulse, blood pressure, and pain. It recommended assessment of psychological distress issues as routine in clinical care (Bultz et al., 2011).

Pituitary adenomas are severe, chronic, multi-organ, and multi-system diseases. Although symptoms can be or are partially relieved with surgery, medications, or other treatment modalities, many patients still develop permanent hypopituitarism and require various hormone replacement therapies throughout their lives (Fukuhara et al., 2022). Symptoms due to tumor compression, complications and side effects due to interventional therapeutic measures, and systemic specific symptoms and manifestations due to excessive and insufficient hormone secretion (Biermasz, 2019) all contribute to psychological distress in patients. For example, infertility is a specific psychological distress associated with prolactinoma (Han et al., 2023; Pereira et al., 2020). Furthermore, patients with non-functioning adenomas develop visual symptoms and hypopituitarism (Gu, 2018), and visual field defects improve after treatment, but regular monitoring of tumor residues is required. If the monitoring is not timely, the risk of secondary surgical resection will be increased when the tumor manifests as a giant adenoma, so even if there are no unique symptoms, it will increase the psychological burden of the patient (Luo, 2020) and cause psychological distress. For patients with Cushing’s syndrome, high healthcare costs (Kreitschmann-Andermahr et al., 2018; Lee et al., 2018) and a decrease in the patient’s quality of life (Page-Wilson et al., 2024) are significant factors contributing to psychological distress. The physical sequelae of patients with acromegaly, including its comorbidities, lead to a decline in physical and mental performance as well as the burden of physical disfigurement, resulting in psychological distress specific to this type of disease (Siegel et al., 2021). It is important to note that the quality of life of patients with pituitary adenomas is significantly influenced by psychological distress, including anxiety and depression, as demonstrated by numerous studies (Sabahi et al., 2024). Consequently, it is imperative to alleviate psychological distress in patients with pituitary adenomas. It is essential to investigate the pertinent factors and mechanisms of action that influence psychological distress in light of the considerations above. The results will serve as a benchmark for the creation of interventions that are specifically designed to mitigate psychological distress in this demographic.

Previous studies have shown that patients’ psychological distress is influenced by various factors, including sociodemographic (Wells et al., 2015), disease-related (Schell et al., 2018), and psychosocial stigma (Huang et al., 2021), social alienation (Felser et al., 2022), loneliness (Gallagher et al., 2021), coping styles (Liu et al., 2018), social support (Al-Ghabeesh et al., 2024), and self-expression (Fang et al., 2024). Although a large number of valuable studies have been accumulated in the field of psychological distress, few researchers have focused on the impact of social support on psychological distress in patients with pituitary adenomas. Social support is the term used to describe the resources individuals acquire through their social relationships, which can alleviate psychological stress, relieve mental tension, and enhance social adaptability (Crudden et al., 2021). This support can be obtained from family, friends, and organizations, and it fosters personal psychological motivation. Being a feature of the environment that affects individuals, social support plays an important role in the perception of general health and determines overall wellbeing. Therefore, social support may be associated with psychological distress in patients with pituitary adenomas. Previous studies have shown that patients with pituitary adenomas often face a lack of social support during medical treatment and during their life (Andela et al., 2018; Biermasz, 2019), and researchers have demonstrated the need to improve psychological and social support for rare diseases such as pituitary disorders in order to optimize treatment. It is also worth noting that some studies have shown that psychological distress (anxiety and depression) has a significant impact on the quality of life of patients with pituitary adenomas (Sabahi et al., 2024). Therefore, investigating the impact of social support on psychological distress is of enormous practical significance to improve the quality of life of patients with pituitary adenomas, given the challenges and opportunities for social support and psychological distress among patients with pituitary adenomas in China.

## 2 Theories and hypotheses

### 2.1 Social support and psychological distress

Chinese scholar Xiao (1994) asserts that individuals can acquire objective and subjective support across various societal dimensions. However, the actual value of social support can only be realized through the comprehensive utilization of these resources. As a result, his research theory divides social support into three dimensions: objective support, subjective support, and support utilization. The psychosocial model of distress identifies socio-cultural factors, personal resources, and stressors as the three primary components of stress (Fang et al., 2024). Numerous studies have demonstrated that social support is a critical environmental factor in mental health and that increased social support effectively reduces stress and adverse emotions that arise from stressful events (McLean et al., 2022). Meanwhile, the Wilson-Cleary model of health-related quality of life states that environmental characteristics such as psychological, social, and economic support play an essential role in overall perceived health. Recent studies have validated that social support is crucial in the self-regulatory process of stress management and is an intrinsic motivator for the recovery of individual resources (Zhang et al., 2022). Social support is offering assistance to others through specific mental or material means to assist them in managing stress. As a positive external source, social support helps patients alleviate psychological distress and improve their quality of life (Al-Dwaikat et al., 2021). It has now been shown that social support for patients and caregivers is associated with psychological distress (Al-Ghabeesh et al., 2024; Fang et al., 2024; Oti-Boadi et al., 2024). Therefore, we believe that social support is closely linked to psychological distress and that higher social support is associated with less psychological distress in patients (Firouzbakht et al., 2020; Zerach and Elklit, 2020). There are fewer reports on the relationship between social support and psychological distress in patients with pituitary adenomas. This study aimed to investigate the relationship between social support and psychological distress in patients with pituitary adenomas and to study the mechanism of their inherent influence.

### 2.2 The intermediary function of self-efficacy

Self-efficacy is an individual’s confidence or belief in their capacity to accomplish behavioral objectives in a specific domain (Bandura, 1995). Self-efficacy is dynamic and can be changed through learning, feedback, and experience (Croy et al., 2020). Self-efficacy is a direct determinant of an individual’s capacity to manage stress and adversity and reflects their confidence in the control and treatment of their illnesses. Specifically, individuals with higher self-efficacy are better equipped to manage their diseases and symptoms (Yuan and Zhao, 2021). Consequently, patients who possess high levels of self-efficacy can manage adversity in a composed manner and prevent adverse effects on their mental health, as opposed to those who possess low levels of self-efficacy. This theoretical proposition has been validated by recent research; numerous studies have identified a negative correlation between self-efficacy and depression (Chen et al., 2020; Hua and Ma, 2022). As an illustration, a survey of cancer survivors in the United States and another study of unemployed youth in China both discovered that lower self-efficacy was correlated with more severe depressive symptoms (Hua and Ma, 2022; Philip et al., 2013). Moreover, numerous studies indicate that interpersonal processes, including social support and interaction, affect self-efficacy. Indeed, evidence suggests a positive correlation between self-efficacy and social support (Chen et al., 2022; Dinh and Bonner, 2023; Yu et al., 2024). Individuals who receive excellent social support generally exhibit increased confidence in their capabilities. Thus, self-efficacy is negatively related to psychological distress and positively associated with social support. Self-efficacy may mediate between social support and psychological distress. However, this mediating relationship has not been rigorously tested in pituitary adenoma patients.

### 2.3 The mediating role of rumination

Rumination is the repetitive contemplation of negative emotional experiences or frustrating situations (Treynor et al., 2003). Nolen-Hoeksema et al. (2008) proposed the reaction style theory in explaining the stress that people face, in which rumination is an adverse reaction style that not only hinders the problem-solving itself but also stagnates the individual in negative emotions and affects the individual’s mental health. Research shows that rumination leads to emotional fatigue, anger, anxiety, insomnia and depression, significantly increasing the risk of mental health disorders (Üzar-Özçetin and Budak, 2024). Recent studies propose that rumination, characterized as a repetitive negative thought process, may correlate with various detrimental psychological outcomes in eating disorders (Palmieri et al., 2021), psychological disorders, and psychiatric symptoms (Mansueto et al., 2021). According to one study, there is a substantial positive correlation between psychological distress and ruminations (Beck et al., 2023). Social support can enhance an individual’s hope and mitigate the stagnation of negative emotions, thereby diminishing rumination. Regarding the correlation between rumination and social support, certain studies have indicated that among Chinese adolescents (Xu et al., 2019), social support is negatively related to rumination. There is a lack of research on rumination in patients with pituitary adenomas.

### 2.4 Chain-mediated effects of self-efficacy and rumination

Self-efficacy can influence cognitive, motivational, affective, and choice processes differently (Bandura, 1978). Individuals with low self-efficacy experience a lack of control over events and feel unable to resolve issues when their psyches are chronically stressed. They are more susceptible to the development of rumination in the presence of adverse cognitive conditions, which can exacerbate depression or negative emotions. When people have high self-efficacy, they are more motivated and active in understanding problems, reflecting on them, and thus recovering in the face of adversity (Andersson et al., 2014). Therefore, we propose that self-efficacy is negatively correlated with rumination.

In summary, social support for patients with pituitary adenomas predicts psychological distress, in which self-efficacy and rumination play a mediating role. Patients’ self-efficacy was negatively correlated with rumination, we hypothesize that self-efficacy and rumination play a chain mediating role in social support and psychological distress.

### 2.5 Theoretical background

The theoretical basis of this study includes the Wilson-Cleary model of health-related quality of life (Wilson and Cleary, 1995) and self-efficacy theory (Bandura, 1978). The Wilson-Cleary model of health-related quality of life, introduced by Wilson and Cleary in 1995, posits that an individual’s or group’s health is determined by a confluence of personal health and environmental factors. In this study, rumination is the symptomatic state in which an individual stagnates in negative emotions after illness, and psychological distress is the individual’s psychological functional state. The health-related quality of life model states that an individual’s symptomatic state influences their functional state; hence, this study concludes that a patient’s rumination influences their psychological distress. The health-related quality of life model also claims that an individual’s symptomatic state and functional state are influenced by both personal and environmental characteristics; in this study, self-efficacy represents a personal characteristic, while social support constitutes an environmental characteristic. Therefore, this study argues that social support and self-efficacy affect individuals’ rumination and psychological distress. Bandura (1978) suggests that self-efficacy formation is influenced by the vicarious experiences of others and through verbal or social persuasion. The indirect experience of others, along with verbal or social persuasion, might be collectively referred to social support. Consequently, this investigation proposed that self-efficacy is impacted by social support. So based on the Health-Related Quality of Life Model and the Self-Efficacy Theoretical Framework, social support, self-efficacy, and rumination influence patients’ psychological distress. Social support and self-efficacy affect patients’ rumination, whereas social support also impacts self-efficacy.

### 2.6 Research hypotheses

**Figure 1:**
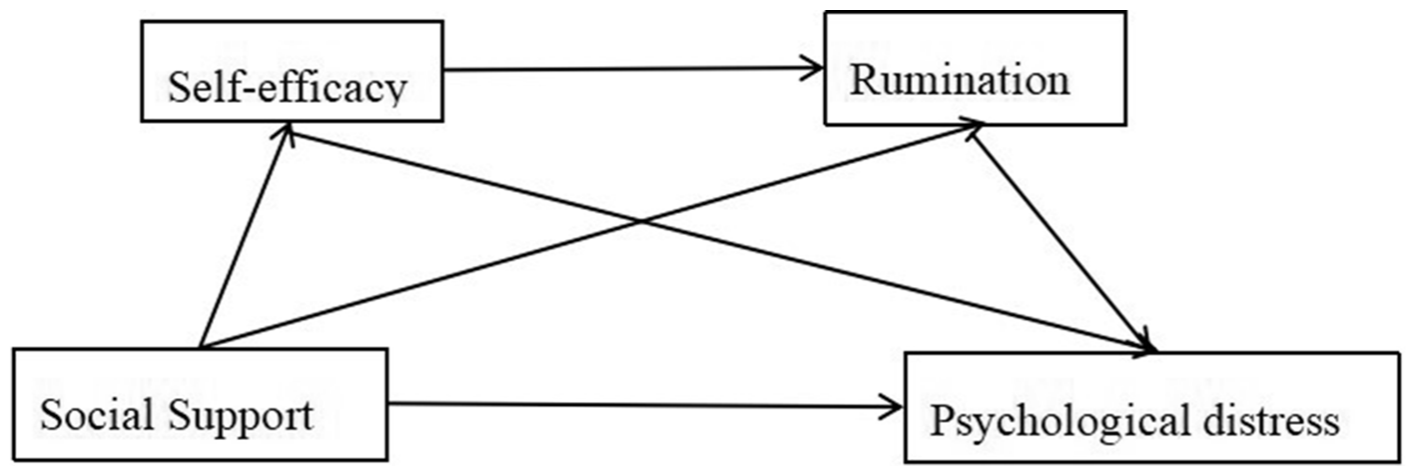
Simplified model of the relationship between the study variables.

The hypothetical model of this study is illustrated in Figure 1, as derived from the preceding theoretical analysis. Considering social support as the independent variable, psychological distress as the dependent variable, and self-efficacy and rumination as the mediating variables, we made the following hypotheses:

*H1*: Social support exhibits a negative correlation with psychological distress in patients with pituitary adenomas.

*H2*: Self-efficacy in patients with pituitary adenomas mediates the relationship between social support and psychological distress.

*H3*: Rumination in pituitary adenoma patients mediates between social support and psychological distress.

*H4*: Self-efficacy and rumination mediate the chain between social support and psychological distress in patients with pituitary adenoma.

### 2.7 Research gap

Psychological distress in patients with pituitary adenomas is an issue that cannot be ignored by clinicians, however, this distress is affected by multiple causes. Al-Ghabeesh et al. (2024) demonstrated that social support mitigates psychological distress in burn survivors, and McLean et al. (2022) established that emotional support correlates with reduced depressive symptoms and enhanced quality of life. Additionally, researchs indicates a negative correlation between perceived social support and psychological distress (Falak et al., 2020; Fang et al., 2024). Guo et al. (2022) demonstrated a substantial negative correlation between social support and rumination. Prior research indicates a correlation between self-efficacy and rumination (Zeng et al., 2021), social support (Al-Dwaikat et al., 2021), and psychological distress (Khalid and Dawood, 2020). The correlation between psychological distress and ruminating has been substantiated in prior research (Beck et al., 2023; Tsai et al., 2021). It is clear that social support, self-efficacy, and rumination all influence psychological distress in patients, but their combined effect on psychological distress remains to be investigated. The purpose of this study was to investigate the relationship between social support and psychological distress in patients with pituitary adenomas and to further explore the chain-mediated role of self-efficacy and rumination. To the best of our knowledge this is the first validation of this chain-mediated model in a pituitary adenoma population, and this study addresses this research gap from both theoretical and empirical perspectives. By doing so, our study attempts to provide a more complete theoretical framework for the impact of social support on psychological distress. Enabling healthcare professionals, families, and the community to provide more resources to enhance support for pituitary adenoma patients. Therefore, the present study aimed to extend the existing literature on social support and psychological distress by examining the mediating role of self-efficacy and rumination.

## 3 Methods

### 3.1 Study design and sample

The necessary sample size was determined utilizing Gpower 3.1 software, with an effect size of 0.15, a power of 0.95, and a significance level of 0.05, resulting in a minimum requirement of 166 participants for nine predictors. The 20% increase resulted from the potential reduction in sample size attributable to non-completion. The poll yielded 500 completed questionnaires.

A total of 520 patients with pituitary adenoma were recruited from the neurosurgery departments of three tertiary general hospitals in Shaanxi Province from March 2024 to December 2024 using convenience sampling. Inclusion criteria: (1) Age between 18 and 65 years; (2) having undergone surgery for pituitary adenoma; (3) signing an informed consent form and voluntarily participating in this study; and (4) being able to communicate and be conscious. Exclusion criteria: (1) Individuals with mental illness who are unable to collaborate; (2) combined with other serious malignant tumors or critical organ insufficiency; (3) combined with infectious diseases such as AIDS or syphilis.

The Second Affiliated Hospital Ethics Committee of Air Force Medical University approved the study protocol (K202410-06). Participants were informed that their involvement in this study was voluntary and confidential, and that they could withdraw without facing any negative consequences. Their data will be utilized solely for research purposes. All subjects gave their consent to participate after receiving the explanation.

### 3.2 Data collection

#### 3.2.1 General demographic characteristics

The demographic characteristics of the questionnaire included gender, age, marital status, fertility status, education, occupation, type of pituitary adenoma, size of pituitary adenoma, and whether the tumor recurred.

#### 3.2.2 Psychological distress scale

In 2020, Sheng (2020) compiled the Chinese psychological distress and needs scale from the Leiden distress and needs scale, developed by Dutch scholars (Andela et al., 2016). The scale comprises 27 items across five dimensions (social function, emotional problems, perception of negative disease, physical and cognitive problems, and sexual function), with a Cronbach’s coefficient of 0.945. The scale is scored on a 5-point Likert scale, with zero representing “not at all” and 4 representing “very much,” and a score > 3 is considered to be severe psychological distress. The equation for the total score is as follows: the actual score of the scale divided by the highest possible score multiplied by 100. The scale is scored on a percentage scale from 0 to 100. Higher psychological distress scores indicate more severe psychological distress. The standardized factor loadings for all entries ranged from 0.642 to 0.944, as demonstrated by the confirmatory factor analysis, suggesting that the structure was valid. The scale’s Cronbach’s alpha in this research was 0.974.

#### 3.2.3 Social support rating scale

The Social Support Rating Scale (SSRS), created by Xiao (1994) in 1994, comprises 10 items across three dimensions: support utilization (three items), objective support (three items) and subjective support (four items). By adding up the scores on each item, we can get the overall score on the scale, which can range from 12 to 66, with higher scores reflecting a greater level of social support. Confirmatory factor analysis revealed that the standardized factor loadings for all items varied from 0.545 to 0.888, signifying robust structural validity. This investigation obtained a scale’s Cronbach’s alpha at 0.894.

#### 3.2.4 General self-efficacy scale

Wang and Liu (2001) revised the General Self-Efficacy Scale, initially created by Schwarzer (1997) in 2001, and produced a Chinese version comprising 10 items. Each has a Likert scale from 1 (completely false) to 4 (completely accurate) for a possible total score of 10–40. Increases in the score indicate a higher level of self-efficacy. Confirmatory factor analysis showed that the standardized factor loadings for all entries ranged from 0.797 to 0.894, indicating good structure validity. The Cronbach’s alpha for the scale in this study was 0.967.

#### 3.2.5 Rumination scale

The Ruminative Responses Scale was compiled by Nolen-Hoeksema (2000). Our scholars Han and Yang (2009) adapted the scale locally, which consists of three dimensions: compelled thinking (five items), reflective thinking (five items), and symptomatic rumination (12 items), and 22 items (e.g., I often think about how lonely I am). Each entry was rated on a 4-point Likert scale, with a score of 1–4 ranging from “never occurs” to “always occurs” and a total score of 22–88, with higher scores indicating more severe rumination. A score of 22–43 is a lower level of rumination, a score of 44 to 65 is a medium level of rumination, and a score of 66 to 88 is a higher level of rumination. Confirmatory factor analysis revealed that the standardized factor loadings for all items varied between 0.654 and 0.889, signifying robust structural validity. The Cronbach’s *α* for this questionnaire in this study was 0.956.

### 3.3 Statistical analysis

Data were analyzed utilizing IBM SPSS version 26.0. Count data were represented as frequencies and percentages, while measured data were expressed as means and standard deviations. Cronbach’s alpha analysis was performed to determine the reliability of the scales in this study. Two independent samples *t*-tests and one-way ANOVA were employed to compare the differences in the four variables among patients with varying characteristics of pituitary adenomas. The Pearson correlation coefficient was utilized to assess the correlation between the variables. We employed IBM AMOS 24.0 to develop and evaluate structural models for hypothesis testing. The proposed model comprised three latent variables (social support, rumination, and psychological distress) and 12 observed variables (subjective support, objective support, support utilization, general self-efficacy, compelled thinking, reflective thinking, symptomatic rumination, social function, emotional problems, perception of damaging disease, physical and cognitive problems and sexual function). This study employed the Bootstrap method, which has been extensively employed in mediation analyses due to its robustness and independence from the assumption of a normal distribution of data, to evaluate the mediating role of self-efficacy and rumination. In order to guarantee the accuracy of the confidence intervals and the stability of the estimates, 500 samples were resampled with 5,000 samples using AMOS 24.0 software. The mediation effect was deemed significant in Bootstrap analysis if the 95% confidence interval did not include zero.

## 4 Results

### 4.1 Descriptive statistics

Five hundred out of 520 patients who participated in the study completed the questionnaire, resulting in a completion rate of 96.15%. Among them, the average age is 48.16 ± 11.15 years; 252 (50.4%) were females, of whom 141 (28.2%) had a college degree or higher. Table 1 presents comprehensive general demographic characteristics.

**Table 1:** Demographic characteristics and comparison of psychological distress among pituitary adenomas (*N* = 500).

Variables	*N* (%)	Psychological distress			*Post-hoc* test	*p*
		Mean ± SD	F or t	*P*		
Gender			−1.440	0.150		
Female	252 (50.4)	48.91 ± 20.57				
Male	248 (49.6)	46.27 ± 20.63				
Age			3.650	*0.006		
18–25 years	11 (2.2)	47.31 ± 31.77			② > ①	0.741
26–35 years	70 (14.0)	49.50 ± 21.94			③ > ②	0.187
36–45 years	101 (20.2)	53.69 ± 16.70			③ > ④	0.004
46–55 years	169 (33.8)	46.19 ± 21.72			⑤ > ④	0.381
56-65 years	149 (29.8)	47.60 ± 20.62			⑤ > ①	0.624
Education level			3.369	*0.018		
Primary and below	87 (17.4)	44.57 ± 18.65			② > ①	0.428
junior high school	171 (34.2)	46.71 ± 21.03			② > ③	0.619
Secondary/high school	101 (20.2)	45.43 ± 22.67			④ > ③	0.013
junior college and above	141 (28.2)	52.02 ± 19.17			④ > ①	0.007
Marital status			0.519	0.595		
Unmarried	22 (4.4)	50.17 ± 28.54				
Married	465 (93.0)	47.35 ± 20.29				
Divorce or separation	13 (2.6)	52.14 ± 17.44				
Occupation			1.894	0.110		
Workers	46 (9.2)	47.71 ± 18.83				
Peasants	242 (48.4)	45.25 ± 21.48				
Freelance work	32 (6.4)	51.36 ± 20.68				
Retired	43 (8.6)	47.67 ± 19.20				
Otherwise	137 (27.4)	50.80 ± 19.75				
Reproductive status			1.763	0.078		
Unborn	103 (20.6)	50.78 ± 23.11				
Having given birth	397 (79.4)	46.77 ± 19.87				
Types of pituitary adenomas			3.253	*0.012		
non-Functional	280 (56.0)	45.52 ± 20.60			② > ①	0.545
Gonadotropin type	77 (15.4)	47.11 ± 17.60			③ > ②	0.038
Adrenal Hormone Type	40 (8.0)	55.39 ± 25.89			③ > ④	0.502
Growth hormone type	68 (13.6)	52.66 ± 21.08			④ > ⑤	0.149
Prolactin type	35 (7.0)	46.50 ± 16.20			⑤ > ①	0.788
Pituitary adenoma size			6.120	**0.001		
Microadenoma	19 (3.8)	38.69 ± 22.35			② > ①	0.064
Macroadenoma	468 (93.6)	47.60 ± 20.25			③ > ②	0.028
Giant adenoma	13 (2.6)	60.33 ± 26.11			③ > ①	0.004
Is there a recurrence			2.868	0.004		
Yes	32 (6.4)	57.64 ± 13.81			① > ②	
No	468 (93.6)	46.91 ± 20.84				

*p < 0.05; **p < 0.001; SD, Standard deviation; N, Number.

An independent samples *t*-test or ANOVA was implemented to compare psychological distress across various general demographics. Psychological distress among pituitary adenoma patients differed significantly (*p* < 0.05) by age, education, type of pituitary adenoma, size, and whether or not the adenoma had recurred.

### 4.2 Common methodological biases

The outcomes of Harman’s one-way test indicated that the standard method bias for the first factor was 33.40%, below the critical threshold of 50% (Howard et al., 2024). In other words, the data is free of any significant standard method bias.

Wu (2010) book “Structural Equation Modeling: Operation and Application of AMOS” asserts that if the absolute value of the skewness coefficient is <3 and the absolute value of the kurtosis coefficient is <8, the variables can be said to adhere to a normal distribution. This study employed SPSS 26.0 to assess the skewness and kurtosis of the data. The findings indicate that the absolute skewness coefficient for all variables is below 3, and the absolute kurtosis coefficient is below 8, suggesting that the variables adhere to a normal distribution.

We conducted a confirmatory factor analysis to determine the standardized factor loadings for all items in the four measures in order to evaluate validity. Initially, the Bartlett sphericity test statistics for the four scales were significant at the 0.1% level, and the KMO values exceeded 0.7, thereby satisfying the criteria for factor analysis. Secondly, the standardized factor loadings for all items in the four measures exceeded the threshold value of 0.5, ranging from 0.545 to 0.944 (Hair et al., 2014). Finally, the average variance extraction value (AVE) for the four scales exceeded 0.5, suggesting that the convergence validity is robust. In order to evaluate the reliability of the four scales in our analysis, we implemented composite reliability (CR). The composite reliability scores of all four scales exceed the recommended value of 0.7. Consequently, the measures utilized in our investigation exhibit a high degree of reliability, in accordance with Table 2.

**Table 2:** Descriptive statistics, normality checks, and reliability and validity test of the scale (*N* = 500).

Variables	Mean	SD	Skewness	Kurtosis	AVE	CR
Social support	32.80	10.52	0.307	−0.833	0.641	0.961
Subjective support	17.81	8.00	0.425	−1.078	0.621	0.929
Objective support	6.80	2.92	0.727	0.039	0.666	0.851
Support utilization	8.20	2.54	−0.371	−0.647	0.667	0.857
Self-efficacy	23.14	8.55	0.194	−0.787	0.747	0.967
Rumination	57.49	13.76	−0.193	−0.350	0.630	0.974
Symptomatic rumination	31.43	8.64	−0.215	−0.583	0.636	0.954
Reflective thinking	13.74	3.61	−0.173	−0.542	0.653	0.904
Compelled thinking	12.32	3.36	−0.087	−0.027	0.593	0.879
Psychological distress	47.60	20.62	0.029	−0.309	0.634	0.979
Physical and cognitive problems	14.51	5.94	−0.077	−0.377	0.522	0.845
Emotional problems	15.74	7.32	−0.016	−0.513	0.694	0.919
Perception of negative disease	8.51	3.40	−0.159	−0.200	0.582	0.846
Sexual function	3.23	2.00	0.137	−0.520	0.645	0.779
Social function	9.42	5.49	0.173	−0.304	0.609	0.884

SD, Standard deviation; AVE, Average variance extracted; CR, Composite reliability.

### 4.3 Correlation analysis

The Pearson correlation analyses results, presented in Table 3, showed that social support was positively correlated with self-efficacy (r = 0.362, *p* < 0.01), negatively correlated with rumination (r = −0.416, *p* < 0.01) and psychological distress (r = −0.399, *p* < 0.01). Self-efficacy was negatively correlated with rumination (r = −0.371, *p* < 0.01) and psychological distress (r = −0.400, *p* < 0.01). Rumination was positively correlated with psychological distress (r = 0.451, *p* < 0.01).

**Table 3:** Correlations among study variables (*N* = 500).

Variables	1	2	3	4	5	6	7	8	9	10	11	12	13	14	15
1. Subjective support	1														
2. Objective support	0.354**	1													
3. Support utilization	0.281**	0.259**	1												
4. Social support	0.926**	0.609**	0.527**	1											
5. Self-efficacy	0.308**	0.248**	0.244**	0.362**	1										
6. Symptomatic rumination	−0.341**	−0.289**	−0.249**	−0.400**	−0.342**	1									
7. Reflective thinking	−0.331**	−0.210**	−0.207**	−0.360**	−0.318**	0.678**	1								
8. Compelled thinking	−0.264**	−0.142**	−0.196**	−0.288**	−0.297**	0.575**	0.607**	1							
9. Rumination	−0.366**	−0.271**	−0.259**	−0.416**	−0.371**	0.946**	0.836**	0.765**	1						
10. Physical and cognitive problems	−0.311**	−0.277**	−0.267**	−0.378**	−0.374**	0.395**	0.365**	0.392**	0.439**	1					
11. Emotional problems	−0.328**	−0.288**	−0.269**	−0.394**	−0.384**	0.403**	0.377**	0.403**	0.450**	0.896**	1				
12. Perception of negative disease	−0.293**	−0.231**	−0.204**	−0.336**	−0.331**	0.348**	0.315**	0.314**	0.378**	0.820**	0.841**	1			
13. Sexual function	−0.302**	−0.207**	−0.206**	−0.337**	−0.343**	0.333**	0.354**	0.349**	0.387**	0.723**	0.734**	0.695**	1		
14. Social function	−0.301**	−0.226**	−0.252**	−0.352**	−0.376**	0.330**	0.328**	0.353**	0.379**	0.780**	0.786**	0.736**	0.840**	1	
15. Psychological distress	−0.337**	−0.278**	−0.272**	−0.399**	−0.400**	0.402**	0.382**	0.404**	0.451**	0.944**	0.956**	0.892**	0.838**	0.901**	1

**p < 0.01.

### 4.4 Chained intermediary model analysis

**Figure 2:**
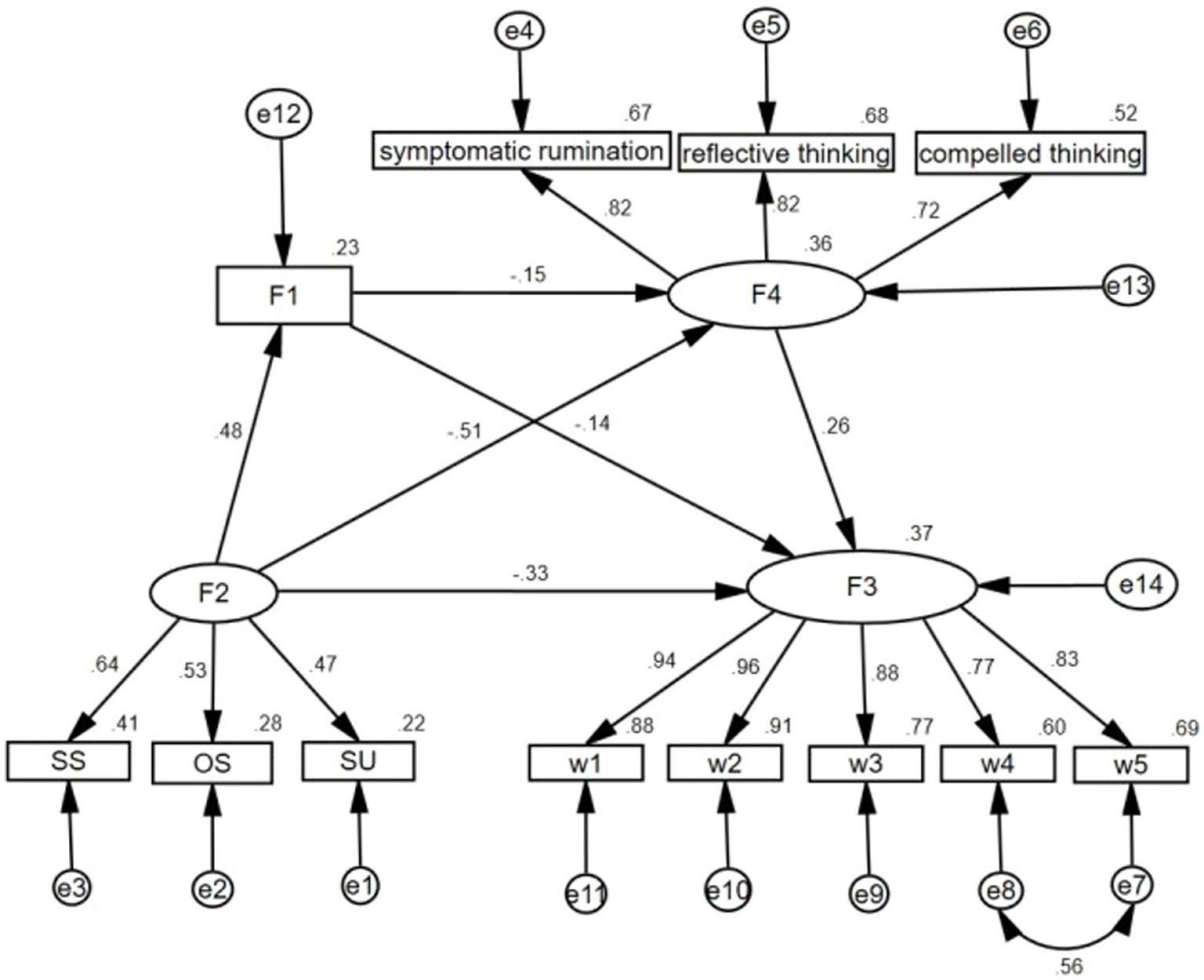
The validated model. F2, social support; F1, self-efficacy; F3, psychological distress; F4, rumination; SS, subjective support; OS, objective support; SU, support utilization; W1, physical and cognitive problems; W2, emotional problems; W3, perception of negative disease; W4, sexual function; W5, social function.

Structural equation modeling examines the pathways linking social support to psychological distress and validates the indirect effects of self-efficacy, rumination, and the chained effects of self-efficacy and rumination. Figure 2 depicts the factor loadings of the corresponding latent variables and the standardized path coefficients of the chain mediation model. The model fit was generally satisfactory, as evidenced by the following values: *χ*^2^/df = 1.119, GFI = 0.982, AGFI = 0.971, NFI = 0.986, IFI = 0.998, CFI = 0.998, RMSEA = 0.015 (Table 4).

**Table 4:** Comparison of model fit for the modified model to the hypothetical model.

Index	*χ* ^2^	df	*χ*^2^/df	GFI	AGFI	NFI	IFI	CFI	SRMR	RMSEA
Fitted model	53.700		1.119	0.982	0.971	0.986	0.998	0.998	0.023	0.015
Fitting criteria	>0.05	48	<5.0	>0.90	>0.90	>0.90	>0.90	>0.90	<0.08	<0.08

*χ2, Discrepancy Chi Square; χ2/df, Chi-square/degree of freedom; CFI, Comparative fit index; RMSEA, Root-mean-square error of approximation; SRMR, Standardized root mean square residual; CI, Confidence interval; GFI, goodness-of-fit index; AGFI, Adjusted Goodness of Fit; NFI, normed fit index; IFI, Incremental Fit Index.*

Regression analyses indicated that social support considerably negatively predicted psychological distress (*β* = −0.328, *p* < 0.001), adversely affected self-efficacy (*β* = 0.484, *p* < 0.001), and positively predicted rumination (*β* = −0.513, *p* < 0.001). Self-efficacy was inversely correlated with rumination and psychological distress (*β* = −0.155, *p* = 0.009; *β* = −0.140, *p* = 0.005). Rumination significantly and positively forecasted psychological distress (*β* = 0.257, *p* < 0.001) (see Table 5).

**Table 5:** Results of multiple regression analysis among variables.

Dependent variable	Predictor variable	*R* ^2^	*β*	SE	*t*	*p*	LLCI	ULCI
Self-efficacy	Social support	0.234	0.484	0.533	6.476	<0.001	0.369	0.597
Rumination	Social support	0.364	−0.513	0.549	−5.524	<0.001	−0.658	−0.373
	Self-efficacy		−0.155	0.049	−2.607	0.009	−0.286	−0.008
Psychological distress	Social support	0.366	−0.328	0.332	−3.763	<0.001	−0.494	−0.167
	Self-efficacy		−0.140	0.027	−2.804	0.005	−0.238	−0.033
	Rumination		0.257	0.042	3.985	<0.001	0.111	0.389

The results of the mediation effect analyses showed that self-efficacy partially mediated the relationship between social support and psychological distress (*β* = −0.068, *p* = 0.004), rumination partially mediated the relationship between social support and psychological distress (*β* = −0.132, *p* = 0.007), and that the interlocking mediation effect between self-efficacy and rumination was also significant (*β* = −0.019, *p* = 0.023). Overall, the total effect of social support on psychological distress was −0.547, of which 60.0% (−0.328) was a direct effect and 40.0% (0.219) was an indirect effect (see Table 6).

**Table 6:** The mediating analysis of self-efficacy and rumination on social support and psychological distress (*N* = 500).

Effect	*β*	SE	LLCI	ULCI	*p*	Contrast effect
Total effect	−0.547	0.053	−0.645	−0.437	<0.001	–
Total direct effect of SS on PD	−0.328	0.085	−0.494	−0.167	0.001	60.0%
Total indirect effect of SS on PD	−0.219	0.046	−0.317	−0.135	<0.001	40.0%
Indirect 1: SS -SE -PD	−0.068	0.025	−0.119	−0.021	0.006	12.4%
Indirect 2: SS -R -PD	−0.132	0.036	−0.215	−0.071	0.001	24.1%
Indirect 3: SS -SE -R -PD	−0.019	0.010	−0.045	−0.003	0.019	3.5%

SS, Social Support; SE, Self-efficacy; PD, Psychological Distress; R, Rumination.

## 5 Discussion

Understanding psychological distress and its underlying psychological mechanisms in patients with surgically treated pituitary adenomas is crucial for effective prevention and intervention of psychological distress. In this study, we validated the relationship between social support and psychological distress in patients with pituitary adenoma through structural equation modeling. Further, we explored the possible pathways linking self-efficacy and rumination. The results of this investigation provide novel empirical insights that enrich our understanding of the mechanisms linking social support and psychological distress. In addition, the results of this study provide theoretical support for effective interventions and prevention strategies to alleviate psychological distress in patients with pituitary adenomas.

The results of this study showed that the postoperative psychological distress of Chinese pituitary adenoma patients was at an intermediate level of 47.60 ± 20.62. This was higher than Sheng Guixiao’s survey of Chinese pituitary adenoma patients, which was 23.89 ± 18.41 (Shen et al., 2021). This analysis is predicated on the fact that the survey subjects were patients with pituitary adenomas who received surgical intervention in tertiary-level hospitals. The patient’s tumors were notably huge, and their symptoms were pronounced. Consequently, the psychological distress level among the patients examined in this study was comparatively elevated. Among the five dimensions, the highest mean score of entries was for the negative disease perception dimension, indicating that patients with pituitary adenoma have a negative perception of the disease. This finding suggests that healthcare professionals should pay appropriate attention to patients’ disease perception. The reason for the negative disease perception of pituitary adenoma patients may be that not all symptoms of pituitary adenoma patients can be improved after active treatment (Andela et al., 2015), and long-term medication and endocrine follow-up may be required. This leads to a low sense of control and fear of disease progression and recurrence. Therefore, clinical staff should provide patients with helpful information about pituitary adenomas to change their negative disease perceptions and positively cope with the disease, thereby reducing psychological distress and enhancing their quality of life.

By analyzing the relationship between demographic information and psychological distress, we found that age, education, type of pituitary adenoma, size of pituitary adenoma, and whether or not the adenoma recurred had a significant effect on the psychological distress of the pituitary adenoma patients themselves. Psychological distress in patients aged 18 to 45 with pituitary adenomas is strongly connected with age. The likely reason for this is the heavier social and family responsibilities that come with age. In addition, pituitary adenomas may cause changes in the patient’s appearance, affecting marriage and sexual life, and patients in this age group are more concerned about the long-term adverse effects of the disease and have higher expectations for recovery. Therefore, we should strengthen long-term follow-up, teach patients self-health management, and provide psychological support to meet their needs. The sample size of patients between the ages of 18 and 25 years in this study was small. As the sample size decreases, the inaccuracy in estimating the overall mean escalates, so compromising the accuracy of the significance test. To ascertain whether patients in this age group experience inferior psychological distress in comparison to those in older age groups, it is necessary to conduct a more extensive investigation. Scholars should conduct additional research on this attribute in the future. Research indicates that patients with larger pituitary adenomas and a surgical history experience have elevated psychological distress. Fallah et al. (2019) have shown that preoperative pituitary adenoma volume is an influential factor in the rate of total resection in transnasal endoscopic surgery and that the larger the preoperative pituitary adenoma volume, the lower the rate of total resection, with large and giant endoscopic total resection rates ranging from 14.3 to 56.4%. In addition, patients who undergo surgery have to go through a lengthy recovery period, and complications such as hypopituitarism and intracranial infection may also occur after surgery. Therefore, healthcare professionals should be concerned about the psychological distress of patients with large tumors and a history of surgery for pituitary adenomas.

Studies have shown that social support has a negative predictive effect on patients’ psychological distress, which is consistent with the results of a previous study (Falak et al., 2020). Patients with higher levels of social support tend to have relatively harmonious family and social relationships and feel more support after the disease, which significantly increases the patient’s confidence in curing it. Therefore, their psychological distress is lower. In the present study, a small number of patients lost their sexual function due to the disease and remained unmarried and childless for life, and they did not have regular social interaction with the opposite sex because of low self-esteem caused by the disease in their adulthood. They belong to a minority group and are unable to receive any form of social support from their families, friends or other peers. As a result, their lives are miserable and psychologically painful. Siegel et al. (2021) conducted qualitative interviews with seven patients with acromegaly and found that patients expressed the need for robust and supportive relationships with medical staff and socio-medical support. Acree et al. (2021) found that by investigating the perceptions of 93 patients with Cushing’s syndrome about their postoperative recovery, the coping mechanisms that patients found to be the most helpful were, first, family and friends, followed by support groups. It can be concluded that patients need to get good social support because good social support can help patient’s better cope with negative emotions and disease risk, promote their recovery, and reduce psychological distress, thereby improving their quality of life. Therefore, it is recommended that healthcare workers should not only pay attention to traditional biochemical indicators in clinical practice and nursing care but also understand the social support ability of patients, help them establish a family support system, and enhance the confidence of patients to overcome the disease, to reduce psychological distress.

The findings suggest that self-efficacy mediates the effect between social support and psychological distress and that self-efficacy directly and negatively predicts psychological distress in patients in the overall structural model constructed. This may be because patients with high social support are more likely to form positive beliefs and increase positive emotions, which can contribute to reducing psychological distress. Self-efficacy is conducive to establishing a healthy and positive mindset in patients, which can improve their confidence in treating the disease (Thornton et al., 2021). In the face of long-term disease distress, the psychological state of patients is often complex and variable. It is prone to anxiety, depression and other emotional problems. At the same time, improving self-efficacy helps patients establish a positive sense of mind, enhance confidence and courage in the face of the disease, and better cope with psychological pressure to reduce emotional distress and maintain a good state of mind. Therefore, it is essential to recognize the critical role of self-efficacy in individual mental health when conducting psychological interventions for patients with pituitary adenoma. Medical staff can use peer education or group counseling to help patients enhance their confidence in overcoming the disease, improve their negative emotions, and reduce their psychological distress.

Meanwhile, our findings suggest that the correlation between social support and psychological distress is mediated by rumination. Interventions targeting the reduction of rumination would be beneficial in improving psychological distress in Chinese patients with pituitary adenomas. A previous study (Oh et al., 2021) showed that patients with lower social support were likelier to experience rumination. Rumination is considered a negative coping style, which makes it difficult for patients to find positive meaning in stressful events, further impacting their mental health (Li et al., 2022). As an essential indicator of cognitive processing in post-traumatic stress, rumination is a necessary path to recovery and growth. Therefore, medical workers should correctly understand the theoretical mechanism, influencing factors and production process of this cognitive processing indicator, pay close attention to high-risk groups, such as patients with low social support and high rumination, and alleviate their rumination through the implementation of personalized interventions to establish a new cognitive schema and psychological structure, achieve positive psychological adjustment after illness, and thus reduce psychological distress.

Finally, this study found a chain-mediated effect of self-efficacy and rumination between social support and psychological distress. Self-efficacy can inversely predict rumination, which is supported by previous empirical findings (Li et al., 2022). The present study found that social support affects psychological distress through self-efficacy and rumination chain mediation. This may be because patients with low social support possess lower levels of self-efficacy, resulting in a lack of confidence to actively regulate adverse events and negative emotions, resulting in enhanced negative emotional experiences. Disorders of inhibitory mechanisms make the individual’s ability to inhibit negative information insufficient, and the individual generates more attention to adverse events and negative emotions, which hinders effective behavior. The individual repeatedly immerses himself in adverse situations, searches for ways to ameliorate negative emotions, and develops rumination. The Emotional Cascade Model (Selby et al., 2013) suggests that rumination of negative emotions increases an individual’s level of negative emotions, which in turn increases attention to emotional stimuli, leading to the production of more rumination and the formation of a snowball-like accumulation of negative emotions, resulting in a tremendous distressing experience for the individual. Overall, enhancing patients’ social support and self-efficacy can reduce patients’ rumination, thereby alleviating their psychological distress. This finding suggests that positive psychological interventions to reduce patients’ psychological distress should prioritize strengthening social support and self-efficacy.

## 6 Significance and limitations of the study

First, this study integrated self-efficacy theory with the Wilson-Cleary model of health-related quality of life to elucidate the process of psychological distress in patients with pituitary adenomas. This integration emphasized social support as an important external resource and self-efficacy as an important internal psychological resource, while identifying rumination as a negative emotional state in which patients live, elucidating their interactions and complex relationships in patients with pituitary adenomas. Second, the understanding of the mechanisms underlying psychological distress was deepened by exploring the mediating effect, which showed that social support was an important predictor of psychological distress in patients with pituitary adenoma, and that self-efficacy and rumination mediated this process and acted together as chain mediators. This study contributes to a deeper understanding of how social support affects patients’ psychological distress through psychological mechanisms, thus providing a theoretical basis for designing effective psychological interventions. Finally, it encourages medical professionals, families and society to emphasize the creation of supportive environments for patients with pituitary adenomas, to improve patients’ self-efficacy in all aspects, to reduce their rumination, to guide them to face health problems positively and optimistically, and to promote the improvement of their quality of life.

This study has the following limitations: firstly, the subjects were patients in tertiary hospitals in Xi’an, Shaanxi Province, which does not reflect that hospitals in Shaanxi Province are in a similar situation. Future studies should expand the sample to hospitals of different levels in different regions to increase the sample’s representativeness. Secondly, the cross-sectional design in the study could not assess the causal relationship of the variables. Longitudinal studies are needed in future studies to further test these variables’ causal relationship. Finally, this study employed a self-reported questionnaire for data collection, resulting in potential subjectivity and bias, constraining the generalizability of the findings.

## 7 Conclusion

This study’s results highlight that social support significantly influences psychological distress via the sequential mediation of self-efficacy and rumination. This study enhances the comprehension of how social support influences the emergence of psychological distress via psychological mechanisms. These findings can inform the development of preventive and intervention strategies designed to alleviate psychological distress and enhance the quality of life for patients with pituitary adenomas.

## Data Availability

The raw data supporting the conclusions of this article will be made available by the authors, without undue reservation.

## References

[ref1] Acree R. Miller C. M. Abel B. S. Neary N. M. Campbell K. Nieman L. K. (2021). Patient and provider perspectives on postsurgical recovery of Cushing syndrome. J. Endocr. Soc. 5:bvab109. doi: 10.1210/jendso/bvab109, PMID: 34195531 PMC8240411

[ref2] Al-Dwaikat T. N. Rababah J. A. Al-Hammouri M. M. Chlebowy D. O. (2021). Social support, self-efficacy, and psychological wellbeing of adults with type 2 diabetes. West. J. Nurs. Res. 43, 288–297. doi: 10.1177/0193945920921101, PMID: 32419665

[ref3] Al-Ghabeesh S. H. Mahmoud M. Rayan A. Alnaeem M. Algunmeeyn A. (2024). Mindfulness, social support, and psychological distress among Jordanian burn patients. J. Burn Care Res. 45, 685–691. doi: 10.1093/jbcr/irad195, PMID: 38126888

[ref4] Andela C. D. Lobatto D. J. Pereira A. M. van Furth W. R. Biermasz N. R. (2018). How non-functioning pituitary adenomas can affect health-related quality of life: a conceptual model and literature review. Pituitary 21, 208–216. doi: 10.1007/s11102-017-0860-4, PMID: 29302835 PMC5849670

[ref5] Andela C. D. Scharloo M. Pereira A. M. Kaptein A. A. Biermasz N. R. (2015). Quality of life (QoL) impairments in patients with a pituitary adenoma: a systematic review of QoL studies. Pituitary 18, 752–776. doi: 10.1007/s11102-015-0636-7, PMID: 25605584

[ref6] Andela C. D. Scharloo M. Ramondt S. Tiemensma J. Husson O. Llahana S.. (2016). The development and validation of the Leiden bother and needs questionnaire for patients with pituitary disease: the LBNQ-pituitary. Pituitary 19, 293–302. doi: 10.1007/s11102-016-0707-4, PMID: 26809957 PMC4858557

[ref7] Andersson L. M. Moore C. D. Hensing G. Krantz G. Staland-Nyman C. (2014). General self-efficacy and its relationship to self-reported mental illness and barriers to care: a general population study. Community Ment. Health J. 50, 721–728. doi: 10.1007/s10597-014-9722-y, PMID: 24676869

[ref8] Bandura A. (1978). Self-efficacy: toward a unifying theory of behavioral change. Adv. Behav. Res. Ther. 1, 139–161. doi: 10.1016/0146-6402(78)90002-4 847061

[ref9] Bandura A. (1995). Self–efficacy in changing societies. New York, NY: Cambridge University Press.

[ref10] Beck S. Whitaker K. Cropley M. (2023). Is rumination associated with psychological distress after a cancer diagnosis? A systematic review. J. Psychosoc. Oncol. 41, 584–609. doi: 10.1080/07347332.2022.2145925, PMID: 36604965

[ref11] Biermasz N. R. (2019). The burden of disease for pituitary patients. Best Pract. Res. Clin. Endocrinol. Metab. 33:101309. doi: 10.1016/j.beem.2019.101309, PMID: 31405752

[ref12] Bultz B. D. Groff S. L. Fitch M. Blais M. C. Howes J. Levy K.. (2011). Implementing screening for distress, the 6th vital sign: a Canadian strategy for changing practice. Psychooncology 20, 463–469. doi: 10.1002/pon.1932, PMID: 21456060

[ref13] Chen X. Qiu N. Chen C. Wang D. Zhang G. Zhai L. (2020). Self-efficacy and depression in boxers: a mediation model. Front. Psych. 11:791. doi: 10.3389/fpsyt.2020.00791, PMID: 33132920 PMC7550717

[ref14] Chen Y. Y. Weng L. C. Li Y. T. Huang H. L. (2022). Mediating effect of self-efficacy on the relationship between social support and self-management behaviors among patients with knee osteoarthritis: a cross-sectional study. BMC Geriatr. 22:635. doi: 10.1186/s12877-022-03331-w, PMID: 35918645 PMC9344710

[ref15] Croy G. Garvey L. Willetts G. Wheelahan J. Hood K. (2020). Anxiety, flipped approach and self-efficacy: exploring nursing student outcomes. Nurse Educ. Today 93:104534. doi: 10.1016/j.nedt.2020.104534, PMID: 32702533

[ref16] Crudden G. Doherty A. Margiotta F. Byrne D. (2021). Investigation of physician burnout and the development of symptoms of anxiety and depression: burnout in consultant doctors in Ireland study (BICDIS). BJPsych Open 7, S17–S18. doi: 10.1192/bjo.2021.103

[ref17] Dai C. Kang J. Liu X. Yao Y. Wang H. Wang R. (2021a). How to classify and define pituitary tumors: recent advances and current controversies. Front. Endocrinol. 12:604644. doi: 10.3389/fendo.2021.604644, PMID: 33815274 PMC8010908

[ref18] Dai W. Zhuang Z. Ling H. Yang Y. Hang C. (2021b). Systematic review and network meta-analysis assess the comparative efficacy and safety of transsphenoidal surgery for pituitary tumor. Neurosurg. Rev. 44, 515–527. doi: 10.1007/s10143-020-01240-3, PMID: 32036504

[ref19] Daly A. F. Beckers A. (2020). The epidemiology of pituitary adenomas. Endocrinol. Metab. Clin. N. Am. 49, 347–355. doi: 10.1016/j.ecl.2020.04.002, PMID: 32741475

[ref20] Dinh T. T. H. Bonner A. (2023). Exploring the relationships between health literacy, social support, self-efficacy and self-management in adults with multiple chronic diseases. BMC Health Serv. Res. 23:923. doi: 10.1186/s12913-023-09907-5, PMID: 37649013 PMC10466814

[ref21] Falak S. Safdar F. Nuzhat Ul A. (2020). Perceived discrimination, social support, and psychological distress in transgender individuals. Psych. J. 9, 682–690. doi: 10.1002/pchj.373, PMID: 32618064

[ref22] Fallah N. Taghvaei M. Sadaghiani S. Sadrhosseini S. M. Esfahanian F. Zeinalizadeh M. (2019). Surgical outcome of endoscopic Endonasal surgery of large and Giant pituitary adenomas: an institutional experience from the Middle East. World Neurosurg. 132, e802–e811. doi: 10.1016/j.wneu.2019.08.004, PMID: 31404693

[ref23] Fang M. Hu W. Xie Z. (2024). Relationships among self-disclosure, social support and psychological distress in caregivers of patients with advanced lung cancer: a mediating model. Eur. J. Oncol. Nurs. 72:102677. doi: 10.1016/j.ejon.2024.102677, PMID: 39033557

[ref24] Felser S. Sewtz C. Kriesen U. Kragl B. Hamann T. Bock F.. (2022). Relatives experience more psychological distress due to COVID-19 pandemic-related visitation restrictions than in-patients. Front. Public Health 10:862978. doi: 10.3389/fpubh.2022.862978, PMID: 35910882 PMC9326244

[ref25] Firouzbakht M. Hajian-Tilaki K. Moslemi D. (2020). Analysis of quality of life in breast cancer survivors using structural equation modelling: the role of spirituality, social support and psychological well-being. Int. Health 12, 354–363. doi: 10.1093/inthealth/ihz108, PMID: 31927594 PMC7322199

[ref26] Fukuhara N. Nishiyama M. Iwasaki Y. (2022). Update in pathogenesis, diagnosis, and therapy of Prolactinoma. Cancers (Basel) 14:604. doi: 10.3390/cancers14153604, PMID: 35892862 PMC9331865

[ref27] Gallagher S. Bennett K. M. Roper L. (2021). Loneliness and depression in patients with cancer during COVID-19. J. Psychosoc. Oncol. 39, 445–451. doi: 10.1080/07347332.2020.1853653, PMID: 33274697

[ref28] Gu Y. (2018). Study on the correlation between symptom distress and quality of life in patients with pituitary adenoma after operation. Suzhou: Suzhou University.

[ref29] Guo T. Zhang Z. Taylor A. Hall D. L. Yeung A. S. Kramer A. F.. (2022). Association of social support with negative emotions among Chinese adolescents during omicron-related lockdown of Shenzhen City: the roles of rumination and sleep quality. Front. Psych. 13:957382. doi: 10.3389/fpsyt.2022.957382, PMID: 36046154 PMC9423767

[ref30] Hair J. F. Black W. C. Babin B. J. Anderson R. E. (2014). Multivariate Data Analysis. 7th Edn. London: Pearson.

[ref31] Han A. J. Fleseriu M. Varlamov E. V. (2023). Symptoms at presentation in conservatively managed patients with non-functioning pituitary adenomas. Hormones 22, 305–309. doi: 10.1007/s42000-023-00444-8, PMID: 36905572

[ref32] Han X. Yang H. (2009). Chinese version of Nolen-Hoeksema ruminative responses scale (RRS) used in 912 college students: reliability and validity. Chin. J. Clin. Psych. 17, 549–551. doi: 10.16128/j.cnki.1005-3611.2009.05.028

[ref33] Howard M. C. Boudreaux M. Oglesby M. (2024). Can Harman’s single-factor test reliably distinguish between research designs? Not in published management studies. Eur. J. Work Organ. Psy. 33, 790–804. doi: 10.1080/1359432X.2024.2393462, PMID: 40101104

[ref34] Hua Z. Ma D. (2022). Depression and perceived social support among unemployed youths in China: investigating the roles of emotion-regulation difficulties and self-efficacy. Int. J. Environ. Res. Public Health 19:676. doi: 10.3390/ijerph19084676, PMID: 35457545 PMC9029286

[ref35] Huang Z. Yu T. Wu S. Hu A. (2021). Correlates of stigma for patients with cancer: a systematic review and meta-analysis. Support Care Cancer 29, 1195–1203. doi: 10.1007/s00520-020-05780-8, PMID: 32951087

[ref36] Khalid A. Dawood S. (2020). Social support, self-efficacy, cognitive coping and psychological distress in infertile women. Arch. Gynecol. Obstet. 302, 423–430. doi: 10.1007/s00404-020-05614-2, PMID: 32458132

[ref37] Kreitschmann-Andermahr I. Siegel S. Gammel C. Campbell K. Edwin L. Grzywotz A.. (2018). Support needs of patients with Cushing's disease and Cushing's syndrome: results of a survey conducted in Germany and the USA. Int. J. Endocrinol. 2018:9014768. doi: 10.1155/2018/9014768, PMID: 30402098 PMC6198616

[ref38] Lee K. Niskanen L. Olson F. Bornheimer R. Maamari R. Neary M. (2018). Budget impact of pasireotide LAR for the treatment of Cushing's disease from a Finnish societal perspective. Value Health 21, S250–S251. doi: 10.1016/j.jval.2018.04.1694, PMID: 40108010

[ref39] Li J. Xue L. Pan H. (2022). Social support and spiritual well-being of patients with esophageal Cancer aged over 50 years: the mediating role of rumination. Front. Psych. 13:805380. doi: 10.3389/fpsyt.2022.805380, PMID: 35308890 PMC8931259

[ref40] Liu Z. Zhang L. Cao Y. Xia W. Zhang L. (2018). The relationship between coping styles and benefit finding of Chinese cancer patients: the mediating role of distress. Eur. J. Oncol. Nurs. 34, 15–20. doi: 10.1016/j.ejon.2018.03.001, PMID: 29784133

[ref41] Luo Y. (2020). The application research of comfortable nursing based on Roy adaptive model in perioperative period of pituitary adenoma patients. Zhengzhou: Zhengzhou University.

[ref42] Mansueto G. Cavallo C. Palmieri S. Ruggiero G. M. Sassaroli S. Caselli G. (2021). Adverse childhood experiences and repetitive negative thinking in adulthood: a systematic review. Clin. Psychol. Psychother. 28, 557–568. doi: 10.1002/cpp.2590, PMID: 33861493

[ref43] McLean C. L. Chu G. M. Karnaze M. M. Bloss C. S. Lang A. J. (2022). Social support coping styles and psychological distress during the COVID-19 pandemic: the moderating role of sex. J. Affect. Disord. 308, 106–110. doi: 10.1016/j.jad.2022.04.036, PMID: 35429530 PMC9005353

[ref44] Nolen-Hoeksema S. (2000). The role of rumination in depressive disorders and mixed anxiety/depressive symptoms. J. Abnorm. Psychol. 109, 504–511. doi: 10.1037/0021-843X.109.3.504 11016119

[ref45] Nolen-Hoeksema S. Wisco B. E. Lyubomirsky S. (2008). Rethinking rumination. Perspect. Psychol. Sci. 3, 400–424. doi: 10.1111/j.1745-6924.2008.00088.x, PMID: 26158958

[ref46] Oh J. M. Kim Y. Kwak Y. (2021). Factors influencing posttraumatic growth in ovarian cancer survivors. Support Care Cancer 29, 2037–2045. doi: 10.1007/s00520-020-05704-6, PMID: 32851485

[ref47] Oti-Boadi M. Andoh-Arthur J. Abekah-Carter K. Abukuri D. N. (2024). Internalized stigma: social support, coping, psychological distress, and mental well-being among older adults in Ghana. Int. J. Soc. Psychiatry 70, 739–749. doi: 10.1177/00207640241227128, PMID: 38327024 PMC11144357

[ref48] Page-Wilson G. Oak B. Silber A. Okeyo J. C. Ortiz N. O'Hara M.. (2024). Holistic burden of illness in patients with endogenous Cushing's syndrome: a systematic literature review. Endocrinol. Diabetes Metab. 7:e464. doi: 10.1002/edm2.464, PMID: 38124436 PMC10782070

[ref49] Palmieri S. Mansueto G. Scaini S. Caselli G. Sapuppo W. Spada M. M.. (2021). Repetitive negative thinking and eating disorders: a Meta-analysis of the role of worry and rumination. J. Clin. Med. 10:2448. doi: 10.3390/jcm10112448, PMID: 34073087 PMC8198834

[ref50] Pereira H. S. Naliato E. C. Moraes A. B. Gadelha M. R. Vieira Neto L. Almeida R. M.. (2020). Body self-image disturbances in women with prolactinoma. Braz. J. Psychiatr. 42, 33–39. doi: 10.1590/1516-4446-2018-0325, PMID: 31314867 PMC6986485

[ref51] Philip E. J. Merluzzi T. V. Zhang Z. Heitzmann C. A. (2013). Depression and cancer survivorship: importance of coping self-efficacy in post-treatment survivors. Psychooncology 22, 987–994. doi: 10.1002/pon.3088, PMID: 22573371 PMC3432138

[ref52] Riba M. B. Donovan K. A. Andersen B. Braun I. Breitbart W. S. Brewer B. W.. (2019). Distress management, version 3.2019, NCCN clinical practice guidelines in oncology. J. Natl. Compr. Cancer Netw. 17, 1229–1249. doi: 10.6004/jnccn.2019.0048, PMID: 31590149 PMC6907687

[ref53] Sabahi M. Yousefi O. Kehoe L. Sasannia S. Gerndt C. Adada B.. (2024). Correlation between pituitary adenoma surgery and anxiety disorder: systematic review and Meta-analysis. World Neurosurg. 187, 184–193. doi: 10.1016/j.wneu.2024.04.154, PMID: 38697260

[ref54] Schell J. T. Petermann-Meyer A. Kloss-Brandstätter A. Bartella A. K. Kamal M. Hölzle F.. (2018). Distress thermometer for preoperative screening of patients with oral squamous cell carcinoma. J. Craniomaxillofac. Surg. 46, 1111–1116. doi: 10.1016/j.jcms.2018.04.022 29789211

[ref55] Schwarzer R. (1997). Optimistic self-beliefs: assessment of general perceived self-efficacy in thirteen cultures. World Psychol. 3, 177–190.

[ref56] Selby E. A. Franklin J. Carson-Wong A. Rizvi S. L. (2013). Emotional cascades and self-injury: investigating instability of rumination and negative emotion. J. Clin. Psychol. 69, 1213–1227. doi: 10.1002/jclp.21966, PMID: 23381733

[ref57] Shen M. Sheng G. Yang Y. Wu C. Ma C. Yang L.. (2021). The distress and needs of Chinese patients with pituitary adenoma: a preliminary survey. Arch. Med. Sci. doi: 10.5114/aoms/143886

[ref58] Sheng G. (2020). Sinicization and application of psychological distress and need scale for pituitary adenoma patients. Suzhou: Suzhou University.

[ref59] Siegel S. Kirstein C. F. Schröder B. Unger N. Kreitschmann-Andermahr I. (2021). Illness-related burden, personal resources and need for support in patients with acromegaly: results of a focus group analysis. Growth Hormon. IGF Res. 61:101422. doi: 10.1016/j.ghir.2021.101422, PMID: 34404019

[ref60] Strickland B. A. Shahrestani S. Briggs R. G. Jackanich A. Tavakol S. Hurth K.. (2021). Silent corticotroph pituitary adenomas: clinical characteristics, long-term outcomes, and management of disease recurrence. J. Neurosurg. 135, 1706–1713. doi: 10.3171/2020.10.Jns203236, PMID: 33962375

[ref61] Thornton C. P. Li M. Yeh C. H. Ruble K. (2021). Self-efficacy in symptom management for adolescents and young adults with cancer: a systematic review. Support Care Cancer 29, 2851–2862. doi: 10.1007/s00520-020-05960-6, PMID: 33403400

[ref62] Treynor W. Gonzalez R. Nolen-Hoeksema S. (2003). Rumination reconsidered: a psychometric analysis. Cogn. Ther. Res. 27, 247–259. doi: 10.1023/A:1023910315561

[ref63] Tsai W. Lee C. S. Monte V. (2021). Comparing the effects of emotional disclosure and peer helping writing on psychological distress among Chinese international students: the moderating role of rumination. J. Clin. Psychol. 77, 1556–1572. doi: 10.1002/jclp.23135 33822363

[ref64] Üzar-Özçetin Y. S. Budak S. E. (2024). The relationship between attitudes toward death, rumination, and psychological resilience of oncology nurses. Semin. Oncol. Nurs. 40:151645. doi: 10.1016/j.soncn.2024.151645, PMID: 38664076

[ref65] Wang K. Liu Y. (2001). Research on the reliability and validity of general self-efficacy scale. J. Appl. Psychol. 1, 37–40. doi: 10.3969/j.issn.1006-6020.2001.01.007

[ref66] Wells M. Cunningham M. Lang H. Swartzman S. Philp J. Taylor L.. (2015). Distress, concerns and unmet needs in survivors of head and neck cancer: a cross-sectional survey. Eur. J. Cancer Care 24, 748–760. doi: 10.1111/ecc.12370, PMID: 26250705

[ref67] Wilson I. B. Cleary P. D. (1995). Linking clinical variables with health-related quality of life: a conceptual model of patient outcomes. JAMA 273, 59–65. doi: 10.1001/jama.1995.03520250075037 7996652

[ref68] Wu M. (2010). Operation and application of structural equation model AMOS. Chongqing: Chongqing University Press.

[ref69] Xiao S. (1994). The theoretical basis and research application of social support rating scale. J. Clin. Psychiatry. 1994, 98–100.

[ref70] Xu W. Jiang H. Zhou Y. Zhou L. Fu H. (2019). Intrusive rumination, deliberate rumination, and posttraumatic growth among adolescents after a Tornadoi: the role of social support. J. Nerv. Ment. Dis. 207, 152–156. doi: 10.1097/nmd.0000000000000926 30807514

[ref71] Yu J. Xie L. Chen S. Fang Z. Zhu L. Zhang H.. (2024). Social support and medication adherence among adult myasthenia gravis patients in China: the mediating role of mental health and self-efficacy. Orphanet J. Rare Dis. 19:143. doi: 10.1186/s13023-024-03145-6, PMID: 38576038 PMC10993533

[ref72] Yuan L. Zhao Z. (2021). Resilience, self-efficacy, social support, and quality of life in patients with skin defects of the lower extremity after flap transplantation. Ann. Palliat. Med. 10, 443–453. doi: 10.21037/apm-20-2432, PMID: 33545776

[ref73] Zeng W. Zeng Y. Xu Y. Huang D. Shao J. Wu J.. (2021). The influence of post-traumatic growth on college Students' creativity during the COVID-19 pandemic: the mediating role of general self-efficacy and the moderating role of deliberate rumination. Front. Psychol. 12:665973. doi: 10.3389/fpsyg.2021.665973, PMID: 33935927 PMC8079774

[ref74] Zerach G. Elklit A. (2020). Attachment and social support mediate associations between polyvictimization and psychological distress in early adolescence. Int. J. Psychol. 55, 380–391. doi: 10.1002/ijop.12590, PMID: 31134627

[ref75] Zhang Y. Zheng P. Dong C. Qichao N. Dong P. Anqi S.. (2022). The association between academic stress, social support, and self-regulatory fatigue among nursing students: a cross-sectional study based on a structural equation modelling approach. BMC Med. Educ. 22:789. doi: 10.1186/s12909-022-03829-2, PMID: 36376814 PMC9664672

